# Ontology-Based Interactive Information Extraction From Scientific Abstracts

**DOI:** 10.1002/cfg.456

**Published:** 2005

**Authors:** David Milward, Marcus Bjäreland, William Hayes, Michelle Maxwell, Lisa Öberg, Nick Tilford, James Thomas, Roger Hale, Sylvia Knight, Julie Barnes

**Affiliations:** 1 Linguamatics Ltd St. John's Innovation Centre Cambridge CB4 0WS UK; 2 AstraZeneca R&D Mölndal Mölndal SE-431 83 Sweden; 3 AstraZeneca R&D Boston 35 Gatehouse Drive Waltham MA 02451 USA; 4 BioWisdom Ltd Babraham Hall Babraham Cambridge CB2 4AT UK

## Abstract

Over recent years, there has been a growing interest in extracting information automatically or semi-automatically from the scientific literature. This paper describes a novel ontology-based interactive information extraction (OBIIE) framework and a specific OBIIE system. We describe how this system enables life scientists to make *ad hoc* queries similar to using a standard search engine, but where the results are obtained in a database format similar to a pre-programmed information extraction engine. We present a case study in which the system was evaluated for extracting co-factors from EMBASE and MEDLINE.


**Comparative and Functional Genomics**
*Comp Funct Genom* 2005; **6**: 67–71.
Published online in Wiley InterScience (www.interscience.wiley.com). **DOI:** 10.1002/cfg.456

**Conference Paper**

### Ontology-based interactive information extraction from scientific abstracts

David Milward[1]*, Marcus Bjäreland[2], William Hayes[3], Michelle Maxwell[4], Lisa Öberg[2], Nick Tilford[4], James Thomas[1], Roger Hale[1], Sylvia Knight[1] and Julie Barnes[4]
[1] *Linguamatics Ltd, St. John's Innovation Centre, Cambridge CB4 0WS, UK*
[2] *AstraZeneca R&D Mölndal, SE-431 83 Mölndal, Sweden*
[3] *AstraZeneca R&D Boston, 35 Gatehouse Drive, Waltham, MA 02451, USA*
[4] *BioWisdom Ltd, Babraham Hall, Babraham, Cambridge CB2 4AT, UK*

**Correspondence to:*
*David Milward, Linguamatics Ltd, St. John's Innovation Centre, Cambridge CB4 0WS, UK.*
*E-mail: david.milward@linguamatics.com*

Received: 17 December 2004
Accepted: 22 December 2004

### Abstract

**Over recent years, there has been a growing interest in extracting information automatically or semi-automatically from the scientific literature. This paper describes a novel ontology-based interactive information extraction (OBIIE) framework and a specific OBIIE system. We describe how this system enables life scientists to make *ad hoc* queries similar to using a standard search engine, but where the results are obtained in a database format similar to a pre-programmed information extraction engine. We present a case study in which the system was evaluated for extracting co-factors from EMBASE and MEDLINE. Copyright © 2005 John Wiley & Sons, Ltd.**

**Keywords:** **oncology; text mining; information extraction; nuclear receptor co-factors**

### Introduction

Information retrieval (IR) systems are designed to find the highest-ranked documents that match a user query, such as a set of keywords. This contrasts with information extraction (IE), which returns relationships, e.g. a table of protein–protein interactions or gene–disease relationships, rather than a ranked set of documents. Traditionally these two techniques have been seen as very different: IR is all about finding documents, IE about finding facts within documents. However, from a user perspective the difference is not so great. The users' aim is primarily to get to information as fast as possible: if a system can get them directly to relevant sentences, then this will save time. If the system can also get them to structured results, appropriately sorted, this can save further time. Interactive information extraction (I2E) is a new concept which combines the interactive querying style of a web search engine with the structured output that is provided by standard IE. This allows scientists to refine queries and explore a set of texts in a similar way to web search, but with the possibility of much more precise search and results.

In the Linguamatics I2E System, the user can start with a standard search for words within a document, then refine this to require the words to be in the same sentence, or in a particular linguistic pattern. The results are output as HTML tables or in a format suitable for database entry. The system pre-indexes documents such as MEDLINE abstracts to allow fast querying. All linguistic processing is done prior to indexing, including 'tokenization' to split a string of characters into individual words, 'sentence splitting' to recognize sentence ends, 'tagging' to recognize parts of speech such as nouns or verbs, and 'chunking' to group words into meaningful units according to their parts of speech.

### The OBIIE framework and system

Conventional IE systems typically allow extraction of syntactic classes (nouns or verbs) and a few

Copyright © 2005 John Wiley & Sons, Ltd.

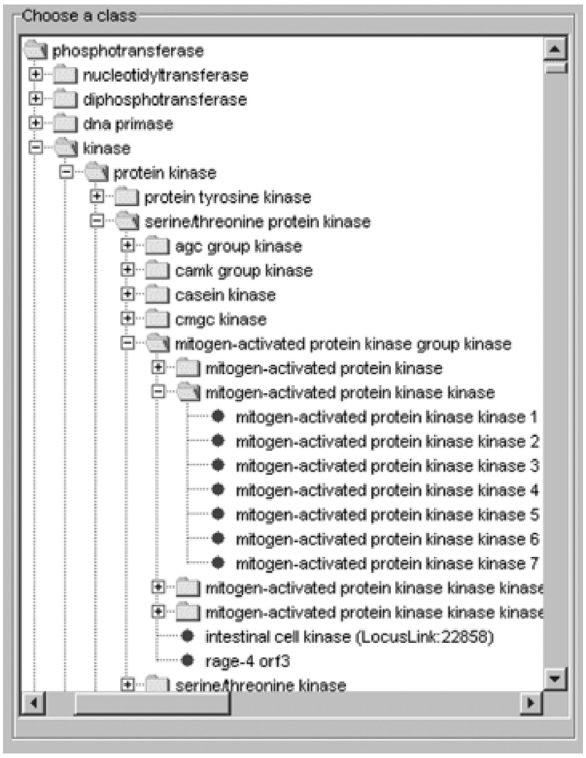

**Figure 1.** Protein Ontology unstructured semantic classes, so called named-entities such as proteins, diseases, or amounts. Named entities may be closed classes formed from an enumerated list e.g. a list of names, or open classes recognized via patterns (e.g. protein spotting routines such as Fukuda 1998). Until recently there has been little use of richer domain knowledge within such IE systems. In ontology-based interactive information extraction (OBIIE), ontologies provide that domain knowledge, enabling the users to interact with the system on a conceptual level without having to know all possible synonyms for a concept. The relationships 'part-of' or 'is-a' provide a basic taxonomy allowing the user to choose particular concepts or families of concepts (see Figure 1). The user can now construct queries using the many thousands of different classes found in typical ontologies.

A scientist may start with a query, such as two keywords in the same document (equivalent to standard keyword search), then refine this to look for two *classes*, e.g. a disease and a protein (co-occurrence within a document), then refine this further to require co-occurrence within a sentence, and finally refine this to look for the protein and disease in a particular syntactic configuration. Incorporation of linguistic constraints is up to the user, and is justified by an increase in precision that is enough to balance any decrease in recall. For example, a query for the word 'RAF' followed by the word 'phosphorylate' would be improved by putting 'phosphorylate' within a so-called 'verb group'. This would then match the text string 'Raf has been shown to phosphorylate', without returning the larger number of false hits you would have got by allowing 'Raf' within, for example, five words of 'phosphorylate'.

The first OBIIE system was constructed by putting together ontologies from BioWisdom with the I2E System. The ontologies incorporated within OBIIE express 'is-a', 'is-a-part-of' (i.e. taxonomic) relationships as well as the 'has synonym' relationship. Ontological concepts can be selected from up to 25 concept types, ranging from genes, proteins, tissues, cells, clinical disorders, symptoms, processes, pathways, drugs, adverse effects, and techniques, technologies, etc. Where appropriate, ontologies can be used as species-specific structures, or as master species-independent ontologies.

As well as defining patterns on the fly, it is possible to reuse existing patterns or pattern templates. Patterns can be organized hierarchically to provide relationship ontologies, so that a user interested in interactions between drugs and proteins can choose the family of 'drug interaction relationships', and obtain alternative phrasings such as the verbs 'activates', 'inhibits', 'blocks' (and their morphological variants) or phrases such as 'is agonist for'.

### Case study: text-mining for nuclear receptor co-factors

In this evaluation we compared the use of two methods. The first consisted of finding a set of abstracts, reading the abstracts, and extracting co-factors from these. The second used the OBIIE tool over a larger set of abstracts, extracting the results, and then filtering these by hand. We were particularly interested in the relative recall between the two methods, and the speed of each method.

Nuclear receptors (NRs) are ligand-dependent transcription factors that typically recruit protein complexes (co-factors) to enhance or repress transcription of target genes. It is believed that several

Copyright © 2005 John Wiley & Sons, Ltd. 

phenomena, such as level of transactivation and tissue specificity of NRs, depend heavily on the specific recruited co-factors. Since NRs are very important drug targets (18 of the 48 known human NRs are targets for registered drugs), the amount of literature on these proteins is rapidly increasing. The aim of this case study was to generate a comprehensive and annotated lists of co-factors for three NRs: androgen receptor (AR) and liver X receptors (LXR) $\alpha$ and $\beta$. AR abstracts were used for 'training' (i.e. query tuning and ontology refinement) and LXR abstracts for testing. The project validated the use of ontology- and linguistic-based text-mining against the previous best practice for obtaining sets of co-factors, based on published or manually generated lists.

For AR we constructed a secondary corpus of 7748 abstracts from MEDLINE and EMBASE by fine-tuning synonym choices. We used two sources for the manual list of co-factors for AR: a list of AR-interacting proteins compiled by Dr Lenore Beitel (Beitel, 2002), and a list constructed by manually examining a subset of abstracts from the secondary corpus containing ~300 abstracts. The abstracts underlying this sub-corpus were further analysed in several iterations with regard to sentences containing information on co-factor recruitment. When we reached sufficient recall (~90%) of extracted relationships, we stopped the iterations. The relationship ontology includes various alternative phrasings and words within larger patterns. Compiling out the embedded alternatives gives a relationship ontology representing 188 distinct patterns. The results of applying the OBIIE system with the relationship ontology are shown in Table 1. The two top reasons why we failed to reach 100% recall was that the secondary corpus did not contain any abstracts with a reference to those symbols (in fact, no abstracts in MEDLINE or EMBASE contained co-occurrences of those symbols and AR), and that references spanned several sentences. Cross-sentence patterns were not used, as they severely affected precision.

Instead of cross-validating the results on the AR secondary corpus, we applied the same query, with the AR synonyms replaced by a selected number of LXR $\alpha$ and $\beta$ synonyms to the whole of MEDLINE (i.e. we did not construct a secondary corpus in this case). The manual list was generated by manual examination of 240 LXR abstracts, but the relationship ontology was not changed based on any of those abstracts. The results of applying OBIIE to extract LXRs from the whole of MEDLINE (the set of MEDLINE abstracts as of 25 November 2003) are displayed in Table 1. One co-factor was missed by the OBIIE system due to a missing pattern in the relationship ontology. The OBIIE system managed to retrieve a co-factor that manual curation missed. For LXRs, recall was 90%. Recall was calculated by dividing the number of co-factors found by OBIIE, divided by the best figure available for the total number of co-factors (the total number of co-factors discovered by either method). Lists of the retrieved co-factors can be found at **http://www.linguamatics.com/obiie/**

The results obtained by the OBIIE system are clearly satisfactory, not only for the recall achieved, but also for the amount of time saved by the automated process. Without risking too many human errors, we estimate that one person can read 100 abstracts in a day. With the tabular output format from the OBIIE system, a domain expert can increase this by an order of magnitude, since only sentences with the NR symbol, a relationship phrase, and a co-factor symbol are displayed.

### Related work and discussion

We have described here the new method of OBIIE for life sciences. There has been much interest in the use of what might broadly be termed 'standard' information extraction techniques in this field (e.g. Blaschke *et al.*, 2002; Craven *et al.*, 1999; De Bruin *et al.*, 2002; Humphreys *et al.*, 2000; Rindflesch *et al.*, 2000; Sekimizu *et al.*, 1998; Thomas

**Table 1.** Results for extraction of NR co-factors

| NR | Abstracts in secondary corpus (*n*) | Abstracts retrieved by OBIIE (*n*) | Co-factors manually retrieved (*n*) | Co-factors retrieved by OBIIE (*n*) | Co-factors found in total (*n*) |
|---|---|---|---|---|---|
| AR | 7748 | 564 | 101 | 100 | 110 |
| LXR $\alpha/\beta$ | N/A | 68 | 9 | 9 | 10 |

Copyright © 2005 John Wiley & Sons, Ltd.

*et al.* 2000) but much less work on the integration of ontologies into the search process. Mädche *et al.* (1999) discuss the use of ontologies as a way to provide information in a canonicalized format to a user or for input into a database, and Todirascu *et al.* (2002) use ontologies for identifying concepts. Although there are precedents for the use of ontologies in information extraction, there are a number of necessary features of any ontology applied in this way. The BioWisdom ontologies used in this case study incorporate a comprehensive list of synonyms, and have a detailed hierarchical structure allowing fine-grained concept distinctions. The domain specific nature of the ontologies also ensures that the synonyms are appropriate to the pharmaceutical domain.

Interactive IE has few precedents. There is some similarity with work on *question-answering systems*. Here NLP and IR techniques are used to obtain the best answer to a question expressed in natural language. In contrast, I2E provides all results for a structured query. There is also some similarity with work that provides an interactive front end on top of the output of a fixed IE system (e.g. Gaizauskas *et al.*, 2001). However, in these systems the result of a new query will always be a subset of the results provided by the original fixed IE query patterns. There are other systems that can be said to be positioned somewhere between document search (IR) and relationship search (IE), but they are typically designed for one specific task, e.g. looking for symbol co-occurrences in sentences. In contrast, in interactive IE there is a natural gradation from document search, via search within sentences, to search for specific relationships within sentences. It is also possible to perform combined searches, e.g. search for relationships, but only in documents containing a particular concept.

A similar examination of co-factors using a co-occurrence-based approach (Albert *et al.*, 2003) searched for tri-occurrences, i.e. entity, relation and entity within the same sentence. Recognition of entities was performed using finite state string matching automata (regular expressions), rather than using linguistic processing. They retrieved fewer co-factors, but it is not appropriate to compare the results directly, since they were working with a smaller corpus, theirs being extracted on 10 September 2001 and ours during the fall of 2003 (21 October for AR, and 25 November for LXRs).

The case study presented here highlights the power of OBIIE in performing systematic textual analysis. The use of synonyms, coupled with the interactive nature of the tool, makes it very quick to engineer queries to accommodate the variety of linguistic forms that phrases take, and hence allows for high recall coupled with good precision. The results for co-factors were remarkably good, with new co-factors discovered by the OBIIE system that had not been in the original manually retrieved set. By exploiting the redundancy inherent in a large corpus, we were able to use relatively specific patterns giving high precision, while still getting recall that rivals that achieved manually. Although the case study in this paper focused on extracting co-factors, the incorporation of other large biomedical ontologies, covering areas of disorders, symptoms, tissues, cells, compounds, biological processes, etc., makes this a flexible tool for use across all parts of pharmaceutical R&D.

### References

Albert S, Gaudan S, Knigge H, *et al.* 2003. Computer-assisted generation of a protein-interaction database for nuclear receptors. *Mol Endocrinol* **17**(8): 1555–1567.

Beitel L. 2002. List of AR-interacting proteins, available from *The Androgen Receptor Gene Mutations Database World Wide Web Server*; **http://ww2.mcgill.ca/androgendb/**.

Biowisdom; **http://www.biowisdom.com**.

Blaschke C, Hirschman L, Valencia A. 2002. Information extraction in molecular biology. *Brief Bioinform* **3**(2): 1–12.

Craven M, Kumlien J. 1999. Constructing biological knowledge bases by extracting information from text sources. *Proceedings of the Seventh International Conference on Intelligent Systems for Molecular Biology (ISMB-99), Sponsored by the International Society for Computational Biology (ISCB), Heidelberg, Germany*. AAAI: Menlo Park, CA; 77–86.

De Bruin B, Martin J. 2002. Literature mining in molecular biology. *European Federation for Medical Informatics (EFMI) Workshop on Natural Language Processing in Biomedical Applications*, Baud R, Ruch P (eds). Nicosia, Cyprus; 1–5.

EMBASE: **http://www.embase.com**.

Fukuda K, Tsunoda T, Tamura A, *et al.* 1998. Toward information extraction: identifying protein names from biological papers. *Proc Pac Symp Biocomput Hawaii* **3**: 705–716.

Gaizauskas R, Herring P, Oakes M, *et al.* 2001. Intelligent access to text: integrating information extraction technology into text browsers. *Proceedings of the Human Language Technology Conference* (HLT2001), San Diego, CA; 189–193.

Humphreys K, Demetriou G, Gaizauskas R. 2000. Two applications of information extraction to biological science journal articles: Enzyme interactions and protein structure. *Pac Symp Biocomput* 502–513.

Copyright © 2005 John Wiley & Sons, Ltd.

Jenssen TK, Laegrid A, Komorowski J, *et al*. 2001. A literature network of human genes for high-throughput analysis of gene expression. *Nature Genet* **28**: 21–28.

Linguamatics Ltd. 2003. Interactive information extraction: white paper; **http://www.linguamatics.com/resources/white_paper_ie.html**.

Mädche A, Staab S, Studer R. 1999. Ontology-oriented information extraction and integration. Workshop on Language Technologies in Information and Knowledge Management. *7th Conference on Computational Linguistics of the German Society for Language Technologies*, Saarbrücken, Germany, 7–8 October.

Medline; **http://www.ncbi.nlm.nih.gov/entrez/query.fcgi?db=PubMed**.

Rindflesch T, Rajan J, Hunter L. 2000. Extracting molecular binding relationships from biomedical text. *1st Conference of the North American Chapter of the Association for Computational Linguistics (NAACL) and 6th Conference on Applied Natural Language Processing (ANLP), Seattle, Washington*. Sponsored by the Association for Computational Linguistics (ACL) 188–195.

Sekimizu T, Park HS, Tsujii J. 1998. Identifying the interaction between genes and gene products based on frequently seen verbs in Medline abstracts. *9th International Workshop on Genome Informatics (GIW'98), Tokyo*. Universal Academy Press: 62–71.

Thomas J, Milward D, Ouzounis C, *et al*. 2000. Automatic extraction of protein interaction from scientific abstracts. *Pac Symp Biocomput* 541–552.

Todirascu A, Romary L, Bekhouche D. 2002. Vulcain — an ontology-based information extraction system. *Natural Language Processing and Information Systems: 6th International Conference on Applications of Natural Language to Information Systems* (NLDB 2002), Stockholm, Sweden; 64–75.

Copyright © 2005 John Wiley & Sons, Ltd. 

## References

[PDF_00328] Albert Sylvie, Gaudan Sylvain, Knigge Heidrun, Raetsch Andreas, Delgado Asuncion, Huhse Bettina, Kirsch Harald, Albers Michael, Rebholz-Schuhmann Dietrich, Koegl Manfred (2003). Computer-assisted generation of a protein-interaction database for nuclear receptors.. Mol Endocrinol.

[PDF_00362] Jenssen T. K., Laegreid A., Komorowski J., Hovig E. (2001). A literature network of human genes for high-throughput analysis of gene expression.. Nat Genet.

[PDF_00382] Sekimizu T, Park HS, Tsujii J (1998). Identifying the Interaction between Genes and Gene Products Based on Frequently Seen Verbs in Medline Abstracts.. Genome Inform Ser Workshop Genome Inform.

[PDF_00387] Thomas J., Milward D., Ouzounis C., Pulman S., Carroll M. (2000). Automatic extraction of protein interactions from scientific abstracts.. Pac Symp Biocomput.

